# Highly Bendable In-Ga-ZnO Thin Film Transistors by Using a Thermally Stable Organic Dielectric Layer

**DOI:** 10.1038/srep37764

**Published:** 2016-11-23

**Authors:** Yogeenth Kumaresan, Yusin Pak, Namsoo Lim, Yonghun kim, Min-Ji Park, Sung-Min Yoon, Hyoc-Min Youn, Heon Lee, Byoung Hun Lee, Gun Young Jung

**Affiliations:** 1 School of Materials Science and Engineering, Gwangju Institute of Science and Technology, 261 Cheomdan-gwagiro, Buk-gu, Gwangju 500-712, Republic of Korea; 2 Department of Advanced Materials Engineering for Information & Electronics, Kyung Hee University, Yongin, Gyeonggi-do 446-701, Republic of Korea; 3 Dongjin Semichem Co. Ltd, Electronic Materials Division, Hwaseong, Gyeonggi-do 445-935, Republic of Korea; 4 Department of Materials Science and Engineering, Korea University, Seoul 136-701, Republic of Korea

## Abstract

Flexible In-Ga-ZnO (IGZO) thin film transistor (TFT) on a polyimide substrate is produced by employing a thermally stable SA7 organic material as the multi-functional barrier and dielectric layers. The IGZO channel layer was sputtered at Ar:O_2_ gas flow rate of 100:1 sccm and the fabricated TFT exhibited excellent transistor performances with a mobility of 15.67 cm^2^/Vs, a threshold voltage of 6.4 V and an on/off current ratio of 4.5 × 10^5^. Further, high mechanical stability was achieved by the use of organic/inorganic stacking of dielectric and channel layers. Thus, the IGZO transistor endured unprecedented bending strain up to 3.33% at a bending radius of 1.5 mm with no significant degradation in transistor performances along with a superior reliability up to 1000 cycles.

For decades, transparent and flexible thin film transistors (TFTs) have drawn much attention and have become the main focus of research in large-area electronics because they can be used in modern electronic applications, such as wearable computers, sensors, photodetectors and rollable displays^1^,^2^,^3^,^4^,^5^,^6^. The key requirement for the realization of flexible electronics is to obtain flexibility with good device performances.

To achieve the flexibility, transistors have to sustain harsh bending strain. Researchers have employed semiconducting polymers as channel materials in flexible organic TFTs (OTFTs) because the weak intermolecular interactions or van der Waals forces between the polymer chains within the organic materials enhance the strain-bearing capability. However, the mobility of the OTFTs is still a challenging issue^1^. With regard to material strategies for enhancing the TFT performance, researchers have focused on amorphous oxide semiconductors (AOSs), especially In-Ga-ZnO (IGZO), because of its high carrier mobility (>10 cm^2^/Vs), high optical transparency and environmental stability^7^,^8^,^9^,^10^,^11^. *Cherenack et al*. used an IGZO channel material for a flexible TFT and demonstrated a good device performance down to a bending radius of 10 mm^12^. Later, *Park et al*. achieved a reliable bending performance up to 5000 cycles in the IGZO TFTs at a 5 mm bending radius (1.25% strain) without any performance degradation^13^. Recently, *Park et al*. demonstrated the flexibility down to 2 mm (corresponding bending strain is 2.5%) with a coplanar IGZO TFTs by adjusting the device structural design (island configuration) as well as its location at the neutral axis plane while being bent^14^. Thus far, the flexible TFTs based on AOSs have employed inorganic dielectrics, such as HfO_2_, ZAO, SiN_X_, and Al_2_O_3_^12^,^13^,^15^,^16^, as gate insulator layers due to their similar crystallinity to the AOS at the interface. However, device performance degradation after repetitive bending cycles is unavoidable due to cracks originating from the non-elastic nature of the stacked inorganic layers. Indeed, there were attempts at creating TFTs based on inorganic semiconductor/organic dielectric layers, including poly (methyl methacrylate) (PMMA), poly (vinyl alcohol) (PVA), and polyimide (PI), to buffer the non-elastic nature^17^,^18^. However, the TFT performances subjected to a certain bending strain were not stable, resulting from the damage to the flexible substrate and/or the organic dielectric layer during the bending cycles.

Generally, the IGZO thin film on an organic dielectric layer was fabricated either by solution processing or sputtering^19^,^20^,^21^,^22^. The solution-processed AOSs have many advantages, including the possibility of large area coating, easy control of the composition ratio and a low fabrication cost; however, they require a high annealing temperature (>300 °C)^19^. Most flexible substrates (PET, PEN, and PI) cannot endure such high temperatures due to substrate deformation, and as a result, the solution-processed AOSs showed the poorer TFT performances. In addition, the mobility of the solution-processed AOSs is relatively low (~5 cm^2^/Vs) compared with those obtained by sputtering^20^,^21^,^23^. The carrier concentration of the sputtered IGZO channel can be modified by controlling the argon (Ar) and oxygen (O_2_) gas flow rate ratio during sputtering^4^,^22^. However, sputtering can incur plasma-induced thermal damage on the underlying organic dielectric layer^18^,^24^. In our preliminary experiment, unwanted wrinkles and roughness on the PMMA dielectric layer was generated during sputtering (supporting information (SI), S1).

To protect the surface of the organic gate dielectric layer against sputtering damage, *B. U. Hwang et al*. deposited an ultrathin Al_2_O_3_ layer on the poly-4vinylphenol (PVP) dielectric layer prior to sputtering the IGZO channel layer^25^, but device flexibility coping with the high bending strain was not demonstrated. Therefore, the stacked non-elastic inorganic layer has to be changed into organic/organic or organic/inorganic stacking to achieve better flexibility. Accordingly, in the case of flexible IGZO TFTs, the inorganic dielectric layer such as Al_2_O_3_ has to be replaced by thermally stable organic dielectric materials which can resist the plasma damage during the IGZO sputtering. In addition, the interface quality between channel layer and dielectric layer plays a critical role in the device performance. In case of the inorganic dielectric layer, there have been several reports that explain the interface quality using positive bias stability (PBS) test^13^,^26^,^27^. However, the interface quality between organic dielectric layer and inorganic channel layer has not been explored.

In this work, we proposed a low-temperature fabrication process of flexible IGZO TFTs on a polyimide (PI) substrate and optimized the transfer characteristics by controlling the Ar:O_2_ gas flow ratio while sputtering. Two different dielectric polymers, PMMA and commercially available poly acrylate based organic material (hereafter referred to as SA7) were employed as a gate dielectric layer. The SA7 polymer was previously used as a buffer layer to achieve a smooth surface and simultaneously to reduce the permeability^13^,^28^,^29^. However, the SA7 has not been used as a gate dielectric layer in TFTs. Thermal properties at the interface between the organic dielectric layer and the IGZO channel layer were investigated. Furthermore, the interface quality was studied by performing the PBS test. Here, IGZO TFTs using the SA7 dielectric layer demonstrated bendability down to a 1.5 mm radius with a bending strain of 3.33% without any significant degradation in the device performances, and maintained superior reliability up to 1000 cycles.

## Results and Discussion

**Figure 1:**
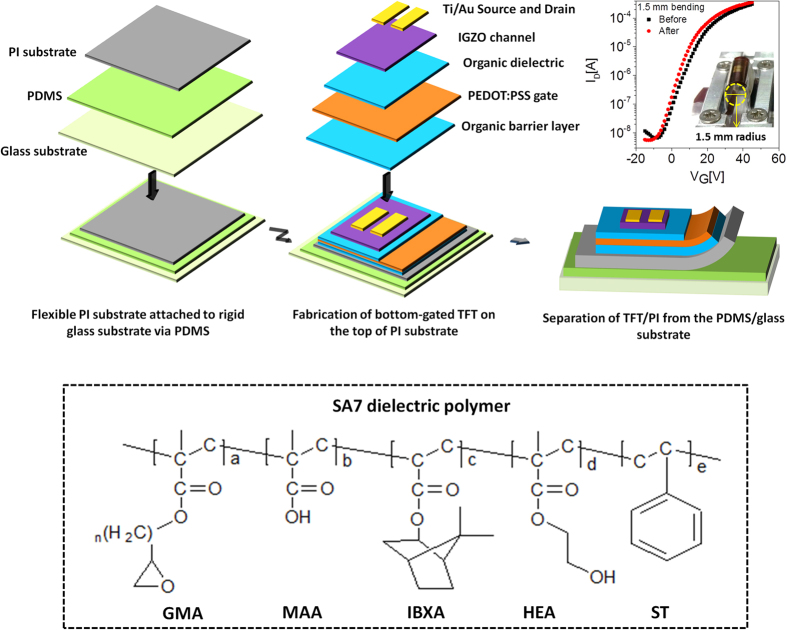
A schematic of fabrication process for flexible bottom-gated IGZO TFT on a polyimide (PI) substrate, which is peeled off from the PDMS layer. A digital image (top right corner) shows the flexible bottom-gated IGZO TFT under the bending test. The chemical structure of SA7 is shown in a dotted box.

Figure 1 demonstrates the schematic of a flexible bottom-gated IGZO TFT fabrication on a 100 μm thick PI substrate, which is explained in detail in the methods section. The digital image of the flexible IGZO TFT under the bending test is shown at the top right corner of Fig. 1. The chemical structure of the commercially available SA7 organic dielectric material is given in dotted box at the bottom of Fig. 1, which is a block co-polymer, consisted of different monomers; glycidyl methacrylate, methacrylic acid, isobornyl acrylate, 2-hydroxyethyl acrylate and styrene. The morphology, i.e., roughness, wrinkles and cracks, of the IGZO semiconducting film plays a critical role in the transistor performance. The sputtered IGZO atoms on the polymer surfaces undergo different kinetic processes including thermal-induced polymer surface modification, surface diffusion of IGZO atoms into the polymer surface, nucleation, island formation and growth^30^. Those kinetic processes govern stress-induced growth, which results in undesirable wrinkles. In our preliminary studies, we sputtered the IGZO film at an Ar:O_2_ gas flow of 100:1 sccm on different gate dielectric layers of commonly used PMMA as a reference and SA7 to study the morphology of the IGZO channel layer (SI, S1). The optical image revealed that there were undesirable wrinkles on the IGZO layer when it was deposited on top of the PMMA. However, in the case of SA7, the deposited IGZO film had a smooth surface with no observable wrinkles. Good thermal stability and better adhesion of SA7 to the IGZO layer could be the main reasons for the smooth IGZO surface after subsequent annealing process. Polymers with a good thermal stability can resist against the plasma-induced thermal damages because the thermal-induced surface modification can be occurred during the sputtering.

**Figure 2:**
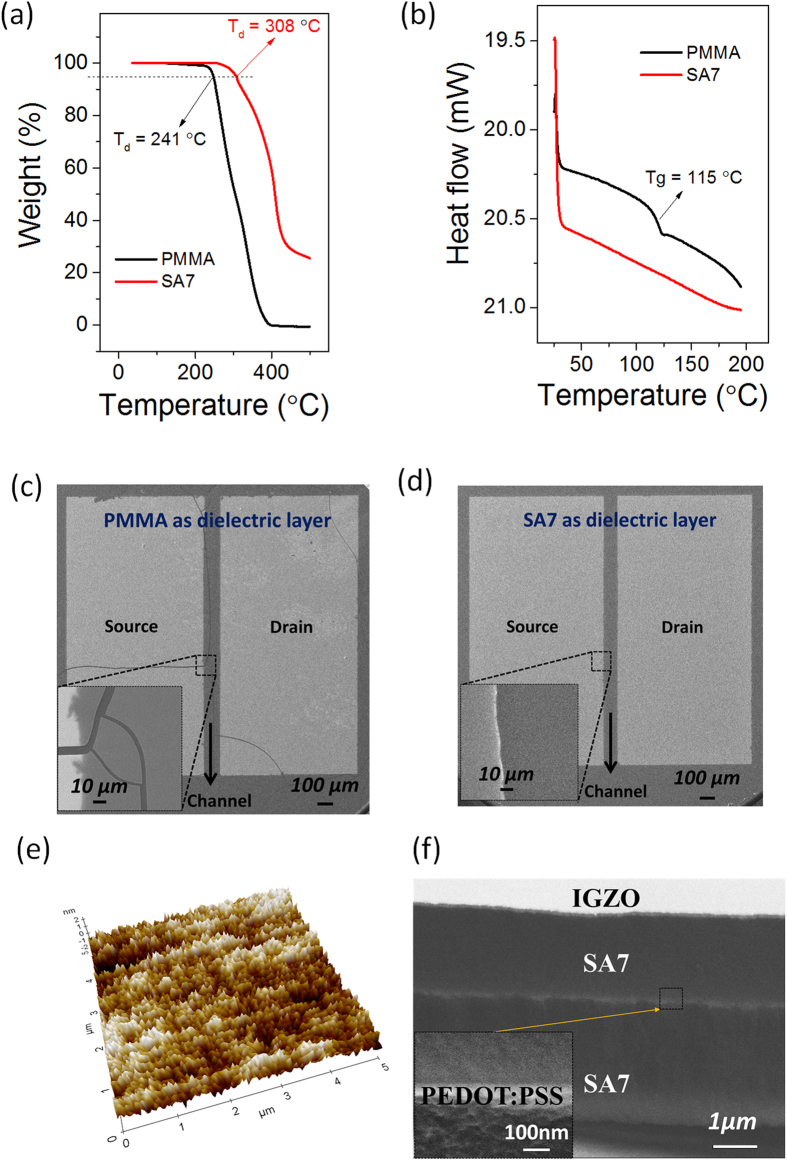
(**a**) The mass loss from the TGA of the PMMA and SA7 under air atmosphere at a heating rate of 5 °C min^−1^. (**b**) DSC thermograms of the PMMA and SA7 in the temperature range of 35 °C ~200 °C. SEM images of the bottom-gated IGZO TFTs on top of (**c**) PMMA and (**d**) SA7 dielectric layer after annealing process at 120 °C. (**e**) An AFM image of the 1.2 μm thick SA7 gate dielectric layer spin-coated on top of a PEDOT:PSS polymer gate electrode. (**f**) A cross-sectional SEM image of the channel region of the bottom-gated IGZO TFT; the inset shows a magnified image of the ~50 nm thick PEDOT:PSS gate electrode sandwiched between the SA7 gate dielectric and barrier layers.

To investigate thermal properties of the PMMA and the SA7, thermogravimetric analysis (TGA) and differential scanning calorimetry (DSC) were utilized. Figure 2a shows the TGA curves of PMMA and SA7 measured in air atmosphere at 5 °C/min. The decomposition temperatures (5% weight loss) for PMMA and SA7 are 241 °C and 308 °C, respectively, which clearly demonstrate that the SA7 has a better thermal stability than the PMMA. Furthermore, DSC was operated in the temperature range from 35 °C to 200 °C to measure the glass transition temperature (T_g_) as shown in Fig. 2b. The PMMA has a T_g_ of around 115 °C but, the SA7 has no endothermic peak in the operating temperature range. At the T_g_, the entangled polymer chains have multiple degrees of freedom and become movable in response to the applied thermal energy. Hence, the IGZO film deposited on the PMMA layer underwent strain (deformation) during the subsequent annealing process at 120 °C, resulting in unwanted cracks within the IGZO film (Fig. 2c). Meanwhile, no cracks were observable in the IGZO film deposited on the SA7 layer (Fig. 2d).

To analyze the interaction of IGZO layer with the underlying organic dielectric layer, FTIR were measured for PMMA, SA7, IGZO/PMMA and IGZO/SA7 (SI, S2). After deposition of IGZO film on top of the SA7 dielectric layer, the intensity at 1150 cm^−1^ (CH_3_ twisting) and 1725 cm^−1^ (C=O of acrylate carboxyl group) were drastically decreased, and slight peak shift and intensity increase were observed at the 600~800 cm^−1^ wavelengths. Those variations are mainly ascribed to the interaction of IGZO atoms with the underlying polymer matrix^31^,^32^,^33^,^34^. However, in case of PMMA, those variations at the 600 to 800 cm^−1^ wavelengths were very small, and there were no observable changes in intensity at the 1150 cm^−1^ and 1725 cm^−1^. Based on above results, the SA7 was selected as a dielectric layer in the following highly bendable IGZO transistor fabrication.

Figure 2e shows the AFM image of the SA7 layer spin-coated on top of the PEDOT:PSS gate electrode with a surface roughness of 0.287 nm. In comparison, the IGZO channel layer deposited on the SA7 revealed a surface roughness of 0.371 nm (SI, S3b). There is no apparent difference in the surface roughness between the two samples. Figure 2f shows the cross-sectional SEM image of the IGZO channel on top of the ~50 nm thick PEDOT:PSS gate electrode, which is sandwiched between the SA7 gate dielectric and barrier layers.

**Figure 3:**
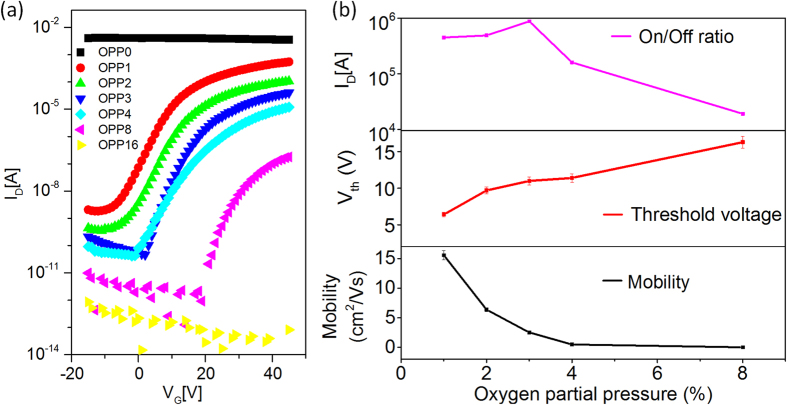
(**a**) The transfer characteristics of the IGZO TFTs with the IGZO active layers deposited at different oxygen partial pressures (OPP) ranging from 1 to 16. (**b**) A comparison of the calculated mobility, threshold voltage and on/off current ratios at different OPPs.

Generally, the IGZO film deposited under only Ar gas flow experiences high oxygen vacancies, which results in a high current level, so that the transistor characteristics cannot be achieved^35^. Therefore, to reduce the oxygen vacancies, the IGZO film is annealed at above 300 °C in ambient condition. However, in this case, the polymeric substrate cannot withstand annealing temperatures above 150 °C. In our experiment, O_2_ gas was also introduced during the sputtering to reduce the oxygen vacancies, which can reduce the subsequent annealing temperature. Seven samples were fabricated at different oxygen partial pressures (OPP, defined in the methods section), which ranged from OPP1 to OPP16, and their transfer characterizations are given in Fig. 3. All the transfer characteristics were measured with the applied drain voltage (V_ds_) of 30 V. Their electrical parameters extracted from the transfer curves are compared in Fig. 3b. The device with IGZO deposited at OPP0 revealed a high current level of 10^−2^ A across the entire bias range, and transistor characteristics were not observed. In the other extreme case of OPP16, the IGZO film became an insulator layer and transistor characteristics were also not observed. The on-current (I_on_) value of the IGZO TFT decreases with an increase in the oxygen partial pressure during sputtering.

Oxygen vacancies in the IGZO channel create gallium and indium positive ions surrounded with free electrons, which are the major charge carriers in the IGZO channel. Therefore, the amount of free electrons can change the transfer characteristics of the TFT along with their electrical properties including the threshold voltage and mobility. By providing oxygen gas during the sputtering, the oxygen vacancies (i.e., free electron carrier concentration) decrease, and the I_on_ value is subsequently reduced. Further, a Hall measurement was performed to directly measure the carrier concentration and resistivity of the IGZO films deposited at different OPP values. It revealed that by increasing oxygen gas flow, the carrier concentration decreased, and the resistivity abruptly increased at OPP3 (SI, S4a). Therefore, a higher gate voltage was required to form an effective channel between the source and drain electrodes, thus resulting in a higher threshold voltage. The mobility also decreased with increasing oxygen gas flow because mobility is directly related to the channel carrier concentration. The devices fabricated at OPP1, OPP2 and OPP3 showed reasonably good transistor performances with an I_on/off_ of >10^5^ and a mobility of >2.5 cm^2^/Vs. In addition, the SA7 organic dielectric layer also exhibited a reasonable gate leakage current of less than 10^−8^ A over the given bias range from −15 V to 45 V.

**Figure 4:**
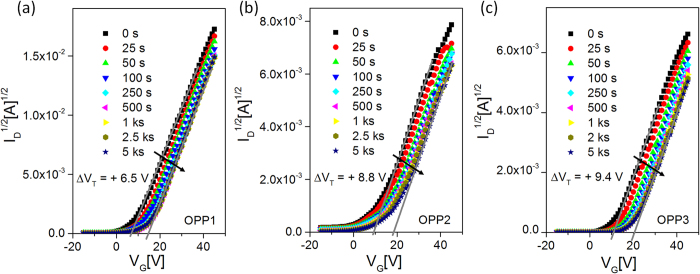
A plot of I_D_^1/2^ [A]^1/2^ vs. V_G_[V] measured at certain time intervals up to 5000 sec for the positive bias stress test at V_GS_ = +40 V, which was applied to the IGZO TFTs fabricated at (**a**) OPP1, (**b**) OPP2 and (c) OPP3.

To analyze the interface quality between the IGZO channel and the SA7 dielectric layer, a positive bias stress (PBS) measurement was performed. With increasing the PBS time, more major charge carriers are likely to be trapped at the channel/dielectric interface, resulting in a higher threshold voltage shift^26^,^27^,^36^. When a certain positive gate bias is applied continuously, the free electrons within the active layer are attracted toward the channel/gate dielectric interface. Then, the defect sites at the low energy level and the deep energy level can trap the free electrons during the PBS. The electrons trapped at the low energy level can be released back to the channel easily in the following voltage sweep. However, the electrons trapped at the deep energy level cannot be released immediately, which reduces the electron carrier concentration within the channel and varies the transistor characteristics. The PBS was conducted with three different IGZO transistors (OPP1, OPP2 and OPP3) by applying a gate bias of +40 V for 5000 sec, and subsequently, the transfer characteristics were measured at different time intervals, as shown in Fig. 4. For all samples, the threshold voltage shifted towards positive as the PBS time increased. Before applying the PBS, the free electrons in the channel were not trapped, and therefore, a channel could be formed immediately under a low threshold voltage. However, as the PBS time increased, more free electrons were trapped to the deep energy level at the interface, and those charges were not released to the channel immediately in the following bias sweeping. Therefore, the net carrier concentration of the channel decreased, resulting in a positive shift of the threshold voltage.

The threshold voltage shift at OPP1 is +6.5 V, which is smaller than those of OPP2 (+8.8 V) and OPP3 (+9.7 V), indicating that the IGZO TFT fabricated at OPP1 has a lower defect trap density compared to the other devices. *Lo et al*. demonstrated the effect of the gas (Ar:O_2_) flow ratio on the trap density at the interface and reported a lower trap density for lower oxygen flow rates^37^. The TFT fabricated at OPP1 exhibited a hysteresis value of ~4 V at 30 V drain bias (SI, S5) along with the best device performance, including a mobility of 15.64 cm^2^/Vs, a threshold voltage of 6.5 V, and a I_on/off_ of 4.5 × 10^5^. Hereafter, the TFTs fabricated at OPP1 condition were utilized for further bending studies. Considering the plasma-induced thermal damage on the organic dielectric layer, our hysteresis value is much lower than that (~10 V) from the IZO/ PVA-co-PMMA bottom-gated transistors^18^.

The subthreshold swing (SS) of our organic dielectric layer-based TFT is high (~3 V/dec) in comparison with the conventional IGZO TFT (~0.5 V/dec) with an inorganic dielectric layer^8^. Generally, high-K dielectric materials (high capacitance) are required to achieve the low SS values (
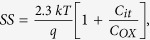
 where *k* is Boltzmann coefficient, *T* is temperature in Kelvin, *q* is electron charge, *C*_it_ = *qN*_*ss*_ is the effective capacitance of trap state at the dielectric/channel interface, *N*_*ss*_ is the trap density at dielectric/channel interface and *C*_*OX*_ is gate oxide capacitance)^10^,^38^. The high SS value of our TFT is ascribed to its low dielectric constant (SA7 = 2.5), which is lower than that of the conventional inorganic dielectric material (SiO_2_ = 3.9, Al_2_O_3_ ≈ 9.5). However, the SS value of SA7-based TFT is lower than that of PMMA-based TFT (~6.5 V/dec, calculated from the transfer characteristics, SI, S6). The *N*_*ss*_ value were also calculated (SI, S6) and revealed that the SA7-based TFT exhibited a lower interface trap density (3.4 × 10^11^ cm^−2^ev^−1^) than that (2.7 × 10^12^ cm^−2^ev^−1^) of PMMA-based TFT owing to the smoother surface and better interface interaction between the IGZO film and the SA7 layer.

**Figure 5:**
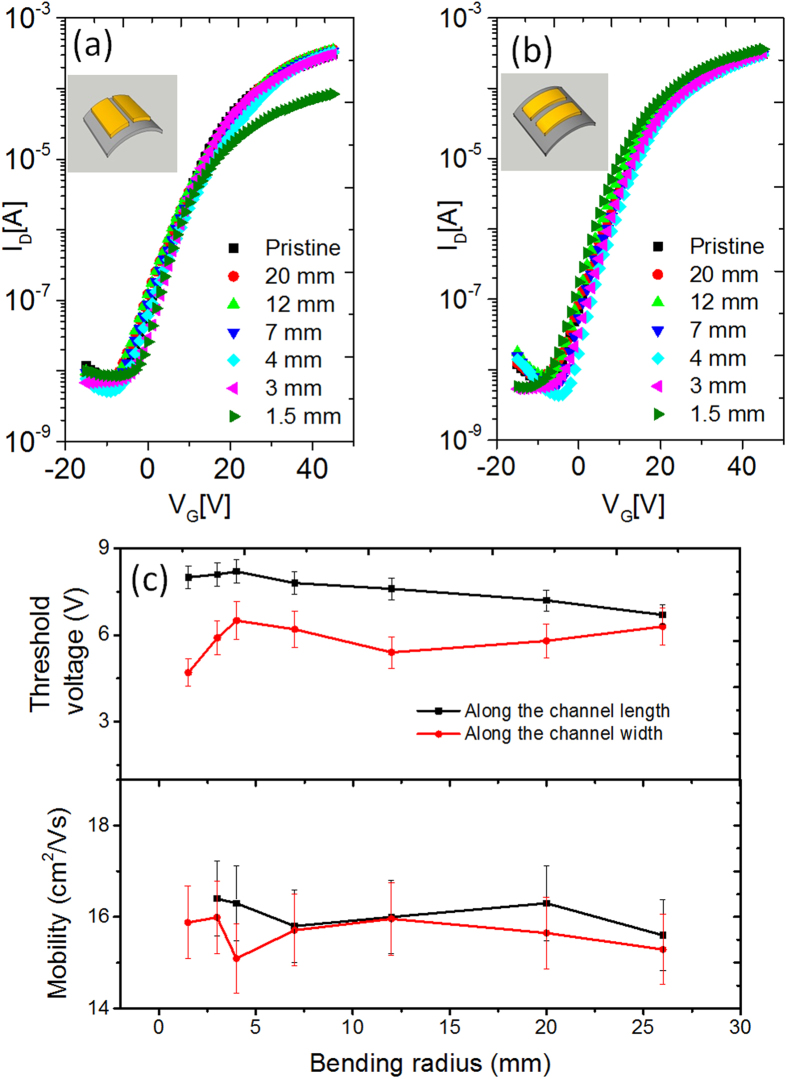
The transfer characteristics of the IGZO TFTs subjected to the bending along (**a**) the channel width direction and (**b**) the channel length direction at various bending radii. The measurements were performed after releasing the bending stress. The inset of Fig. 5a and b shows the schematic of the device bent along the channel width and the channel length direction, respectively. (**c**) A comparison of the mobility and threshold voltage with respect to the bending radii in the two bending cases.

Two IGZO TFTs fabricated at OPP1 were subjected to bending; one device was bent along the channel width direction (inset of Fig. 5a), and the other device was bent along the channel length direction (inset of Fig. 5b) with different bending radii ranging from 20 mm to 1.5 mm. All the bending experiments were performed in ambient condition with a bending tester (SI, S7). The transfer and output characteristics were measured after releasing the bending stress. The electrical parameters, including the mobility and threshold voltage, were extracted from the transfer curves at various bending radii, and their results are given in Fig. 5c. In the case of bending along the channel length direction, the device exhibited almost no degradation in the transistor performance; this result was even applicable to the case of 1.5 mm bending radius, corresponding to a bending strain of 3.33%. When they were bent along the channel width direction, the transfer characteristics were maintained until the sample was bent at a 3 mm bending radius; however, the I_on_ value decreased as the bending radius was decreasing further (1.5 mm).

The transfer characteristics were also measured while being bent along the channel length or channel width direction (SI, S8). Both samples exhibited good bending stabilities without significant performance degradation at a bending radius as low as 4 mm, which corresponds to a bending strain of 1.25%. However, below 4 mm bending radius, the polymer dielectric layer will undergo stretching and thinning, resulting in an increase of the threshold voltage.

**Figure 6:**
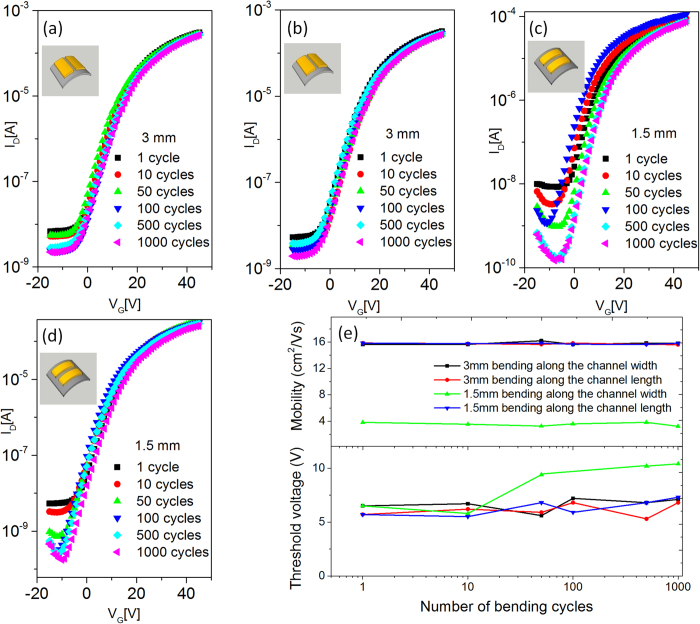
Transfer characteristics of IGZO TFT after different bending cycles along the channel width direction at a bending radius of (**a**) 3 mm and (**b**) 1.5 mm as well as along the channel length direction at a bending radius of (**c**) 3 mm and (**d**) 1.5 mm. (**e**) Comparison of the mobility and threshold voltage with the bending cycles in the four cases. The bending was performed in ambient conditions, and the measurements were performed after releasing the bending stress.

The surfaces of TFTs at pristine state, and while being bent at 3 mm and 1.5 mm radius were observed using an optical microscope (SI, S9, S10). There are no visible cracks along the channel with 100 × magnification while being bent at the 3 mm bending radius in both directions. However, when the device was bent along the channel width at a bending radius of 1.5 mm, nanoscale cracks were visible running along the channel width direction (SI, S9c). Once the bending stress was released, those nanoscale cracks were no longer visible with a microscope (SI, S9d). These nanoscale cracks, which were running perpendicular to the channel length, were responsible for the decrease in the I_on_ current. The mobility was also seriously reduced to 3.83 cm^2^/Vs after the first bending, but it remained constant for the following bending cycles, as shown in Fig. 6e. In comparison, when the device was bent along the channel length, the cracks ran parallel to the channel length direction (SI, S10c). In this case, the TFT demonstrated reliable transfer characteristics even at a 1.5 mm bending radius without any performance degradation in terms of mobility and threshold voltage.

To study the effect of the bending cycles on the TFT performance and their crack density, we fabricated a set of four TFTs. Two TFTs were used for bending along the channel length direction, and the other two TFTs were used for bending along the channel width direction. Their transfer characteristics with respect to the bending cycles (from 1 to 1000 cycles) at a bending radius of 3 mm and 1.5 mm are shown in Fig. 6a–d. In the case of 3 mm bending radius, the TFTs were stable, showing no variation in transistor performances even after 1000 bending cycles regardless of the bending direction. In the case of the 1.5 mm bending radius, there was no variation in mobility with bending cycles for both bending directions, as shown in Fig. 6e, but the threshold voltage increased with the bending cycles only when the sample was bent along the channel width. In contrast, a negligible variation in the threshold voltage was observed when the sample was bent along the channel length. The I_off_ current decreased with the bending cycles along both bending directions. Interestingly, the transistor bent along the channel length direction exhibited a higher on/off current ratio after 50 cycles as a result of the resultant lower I_off_ value, and it was then maintained for the following 500 to 1000 bending cycles. The optical images of the transistor channel obtained during the 1000 bending cycles along the channel length direction at a 1.5 mm bending radius showed no noticeable cracks (SI, S11).

We assume that the moisture trapped at the cracks during the repeated bending test in ambient conditions may act as additional charge trapping sites and can lower the I_off_ value. To clarify the effect of moisture on the I_off_ current during the bending cycles, the sample, which was already subjected to 1000 bending cycles along the channel length direction in ambient condition, was placed in a glove box (nitrogen atmosphere), and measured the transfer characteristics at different time intervals for a week (SI, S12a). Interestingly, the transfer characteristics returned to the initial I_off_ current level after three days. We also performed a 100 bending cycle test along the channel length at a 1.5 mm bending radius inside the glove box and revealed no significant decrease in the I_off_ current (SI, S12b). Furthermore, the device exhibited excellent stability when it was placed in nitrogen atmosphere, even after 45 days (SI, S13).

In summary, we fabricated flexible IGZO TFTs on PI substrates with the help of SA7 organic material as dielectric/barrier layers. The oxygen vacancies (i.e., carrier concentration) within the IGZO channel were optimized by supplying oxygen gas while sputtering the IGZO active film, and thus, subsequent annealing of the IGZO film to reduce the oxygen vacancies could be performed even at 120 °C, a suitable temperature that the underlying polymer substrate could withstand. The device fabricated at an Ar:O_2_ ratio of 100:1 revealed excellent transistor characteristics with a mobility of 15.64 cm^2^/Vs, a threshold voltage of 6.4 V and an on/off current ratio of 4.5 × 10^5^. Additionally, the TFT revealed a good bias stability with a small voltage shift of +6.5 V during the positive bias stress test at V_GS_ = +40 V for 5000 sec. The IGZO transistors sustained harsh bending cycle tests with a bending strain of 3.33% (1.5 mm radius) without any performance degradation and demonstrated stable transistor characteristics even after 1000 bending cycles, thus showing the unprecedented flexibility of these inorganic-based transistors.

## Methods

Bottom-gated IGZO TFTs were fabricated on a 100 μm thick PI substrate. To avoid undesirable crack formation during the fabrication, the thin flexible PI was attached to a rigid glass substrate using an elastic poly-dimethyl siloxane (PDMS, Sylgard™ 184, Dow Corning) interlayer. Owing to the adhesive nature of PDMS, the PI substrate can be attached to the PDMS/glass substrate tightly at an annealing temperature of 120 °C and at a vacuum pressure of 3 × 10^−6^ mbar during the IGZO sputtering. To achieve a smooth surface and a considerable reduction in the permeability, 2 μm thick SA7 (TR-8857-SA7, dielectric constant = 2.5, Dongjin Semichem Co. Ltd, South Korea) was spin-coated as a barrier layer on the PI substrate and annealed at 120 °C for 2 hrs^13^. An organic gate electrode made from poly(3, 4-ethylenedioxythiophene):poly(styrenesulfonate) (PEDOT:PSS, <50 nm thick) was spin-coated on top of the barrier layer and treated with methanol to enhance the conductivity^39^. A 1.2 μm thick SA7 gate dielectric layer was spin-coated on top of the PEDOT:PSS and subsequently annealed at 120 °C for 2 hrs. A 50 nm thick n-type IGZO active layer was deposited by DC sputtering using an IGZO (In_2_O_3_:Ga_2_O_3_:ZnO = 1:1:1 atomic ratio) target at a pressure of 3 × 10^−6^ mbar at different oxygen partial pressures (OPP), which was defined by OPP (%) = P_O2_/(P_O2_ + P_Ar_) × 100^40^, the O_2_ flow rate was varied from 1.01 sccm to 19 sccm with an Ar flow rate at 100 sccm under a constant chamber pressure of 5 mTorr.

To see the effect of the OPP on the device performance, we deposited IGZO film at seven different OPP conditions that varied from 0%, 1%, 2%, 3%, 4%, 8% and 16%; we named them as OPP0 (for the IGZO sputtering at an oxygen partial pressure of 0%), OPP1, OPP2, OPP3, OPP4, OPP8 and OPP16, respectively. After the deposition of the active layer, source and drain (S/D) electrodes (Ti/Au: 5/50 nm) were deposited using a shadow mask with a channel length and width of 100 μm and 2 mm, respectively. Prior to the detachment of the TFTs from the PDMS/glass supporting substrate, the whole setup was annealed at 120 °C. The TFT easily peeled off at the interface between the PI and PDMS with a small external force. The IGZO channel region, including the source/drain electrodes, was observed using an optical microscope before and after the separation from the PDMS/glass substrate (SI, S14).

Thermal properties of the PMMA and SA7 were measured using TGA (TGA 4000, PerkinElmer) and DSC (DSC 4000, PerkinElmer). FTIR spectra were recorded using a FTIR Spectrometer (Varian 660-IR, Varian) in the range 600~2250 cm^−1^. The surface morphology and roughness of the SA7 organic dielectric and the IGZO active layer were analyzed using an atomic force microscope (AFM, XE-100 and Park Systems). A scanning electron microscope (SEM, JSM-7500F and JEOL) was used to analyze the cross-sectional view of the fabricated TFT. A Hall measurement was used to measure the carrier concentration of the IGZO channel. All electrical characterizations were performed using a semi-conductor parameter analyzer (Keithley 4500 C) inside a glove box at room temperature.

## Additional Information

**How to cite this article**: Kumaresan, Y. *et al*. Highly Bendable In-Ga-ZnO Thin Film Transistors by Using a Thermally Stable Organic Dielectric Layer. *Sci. Rep.* **6**, 37764; doi: 10.1038/srep37764 (2016).

**Publisher's note:** Springer Nature remains neutral with regard to jurisdictional claims in published maps and institutional affiliations.

## Supplementary Material

Supporting Information
