# Supplementary material for: Highly Bendable In-Ga-ZnO Thin Film Transistors by Using a Thermally Stable Organic Dielectric Layer
Source: Sci Rep. 2016 Nov 23;6:37764. doi: 10.1038/srep37764 (PMC5120347; doi:10.1038/srep37764)
Supplement: Supporting Information [file srep37764-s1.pdf]

Supporting Information

Highly Bendable In-Ga-ZnO Thin Film Transistors by Using a Thermally Stable Organic Dielectric Layer

Yogeenth Kumaresan, Yusin Pak, Namsoo Lim, Yonghun kim, Min-Ji Park, Sung-Min Yoon, Hyoc-Min Youn, Heon Lee, Byoung Hun Lee, and Gun Young Jung*

S1. Optical microscope images of sputtered IGZO thin films on different dielectric polymers:

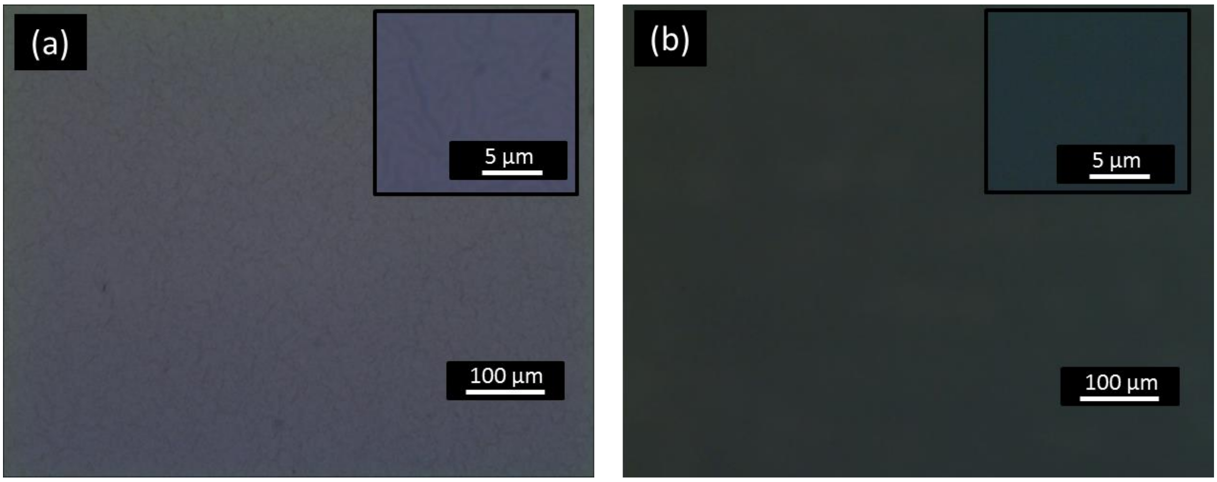

Figure S1. Optical microscope images of the IGZO thin film deposited on different dielectric polymers: (a) PMMA and (b) SA7. The inset shows 100 x magnified image.

Organic dielectric polymers, such as poly-methyl methacrylate (PMMA) and commercially available acryl based polymer (SA7), were spin-coated on a polyimide (PI) substrate and annealed at 120 °C for 2 hrs. Then, the IGZO channel layer was deposited on top of the organic dielectric layers by sputtering at an Ar:O_2 gas flow ratio of 100:1. Optical microscope images show that there are undesirable wrinkles in the IGZO layer deposited on top of the PMMA, as shown in Fig. S1a. However, the surface of IGZO thin film deposited on the SA7 was smooth without any wrinkles, as shown in Fig. S1b.

S2. FTIR spectra of SA7, PMMA, IGZO/SA7 and IGZO/PMMA layers after annealing at 120 ^{o}C:

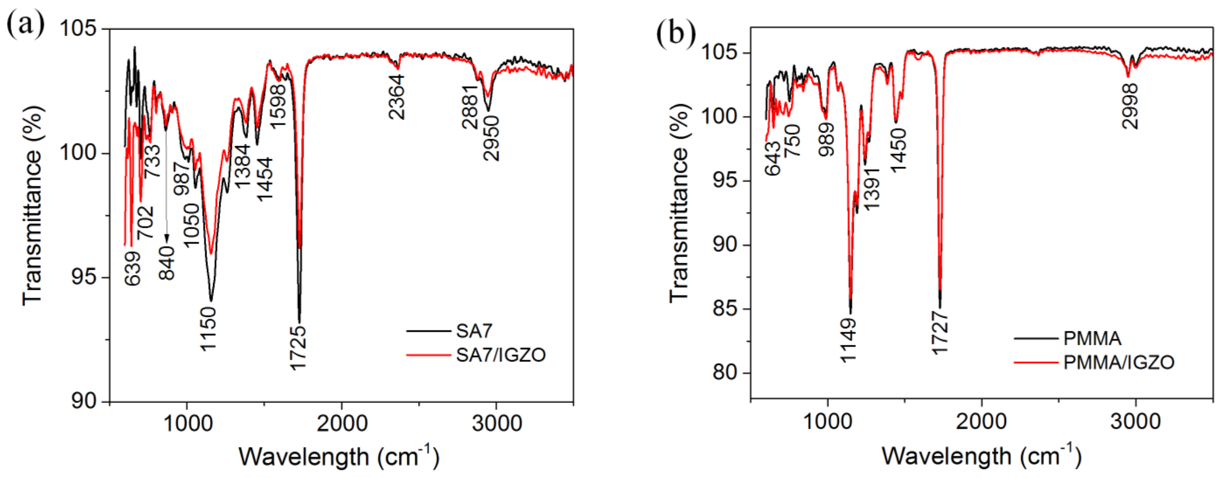

Figure S2. FITR spectra of (a) the SA7 dielectric layer and IGZO/SA7 film and (b) the PMMA dielectric layer and IGZO/PMMA film.

FTIR spectra of the SA7 and IGZO/SA7 are given in Fig. S2a. In SA7, the peaks were observed at 840 cm^{-1} (CH_2 rocking), 987 cm^{-1} (methacrylic acid), 1050 cm^{-1} (C-O-C stretch), 1150 cm^{-1} (CH_3 twisting), 1384 cm^{-1} (α methyl group), 1454 cm^{-1} (bending vibration of C-H), 1598 cm^{-1} (C=C stretching), 1725 cm^{-1} (C=O of acrylate carboxyl group), 2364 cm^{-1} (CO_2 peak), 2881 cm^{-1} (C-H alkane stretch) and 2950 cm^{-1} (C-H bond stretch vibration). In comparison, FTIR spectra of the PMMA and IGZO/PMMA are shown in Fig. S2b. In PMMA, the peaks were observed at 989 cm^{-1} (characteristic vibration of PMMA), 1149 cm^{-1} (C-O-C stretch), 1391 cm^{-1} (α methyl group), 1450 cm^{-1} (bending vibration of C-H), 1727 cm^{-1} (C=O of acrylate carboxyl group), and 2998 cm^{-1} (C-H bond stretch vibration)[1]. After depostion of 30 nm thick IGZO film on the SA7 dielectric layer, the intensity at 1150 cm^{-1} (CH_3 twisting) and 1725 cm^{-1} (C=O of acrylate carboxyl group) were drastically decreased, and slight peak shift and intensity increase were observed at the 600~800 cm^{-1} wavelengths. Genreally, the observed bands at the 600 to 800 cm^{-1} wavelengths are originated from the interaction of IGZO atoms with the underlying polymer matrix[2-5]. However, in case of PMMA, those variations at the 600 to 800 cm^{-1} wavelengths were very small, and there were no observable changes in intensity at the 1150 cm^{-1} and 1725 cm^{-1}. These results clearly indicate the improved interaction of IGZO film to the underlying SA7 dielectric layer, compared to the PMMA dielectric layer.

S3. AFM images of the SA7 dielectric layer and IGZO thin film deposited on the SA7 layer:

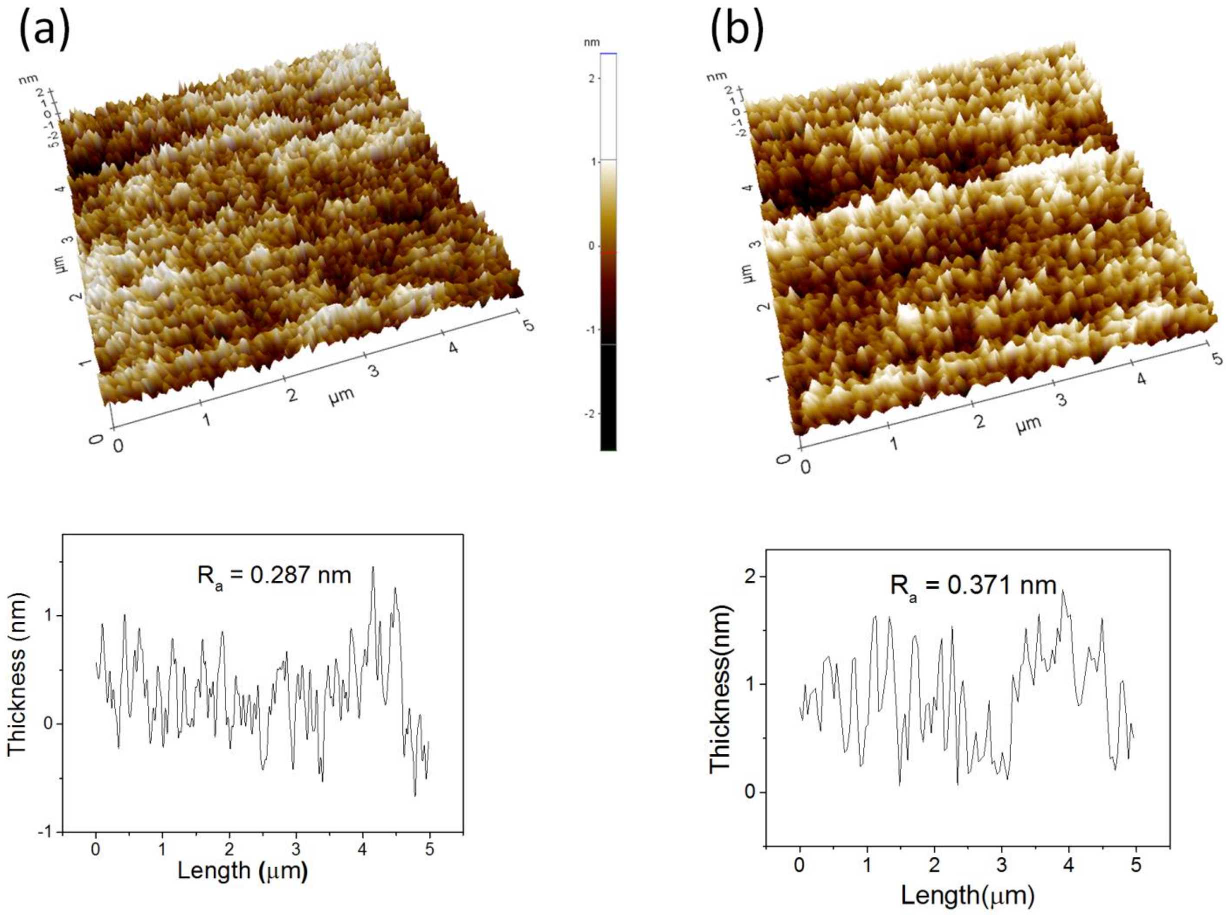

Figure S3. AFM images of (a) the 1.2 µm thick SA7 dielectric layer spin-coated on the PEDOT:PSS gate electrode and (b) the IGZO channel layer deposited on top of the SA7 layer.

AFM images reveal that there is no significant difference in roughness (R_a) between the SA7 dielectric layer (0.287 nm) and the IGZO thin film (0.371 nm) deposited on it. Thus, a high quality IGZO thin film with a smooth and wrinkle-free surface could be achieved.

S4. Electrical properties of the IGZO film deposited at different OPP values:

(a)

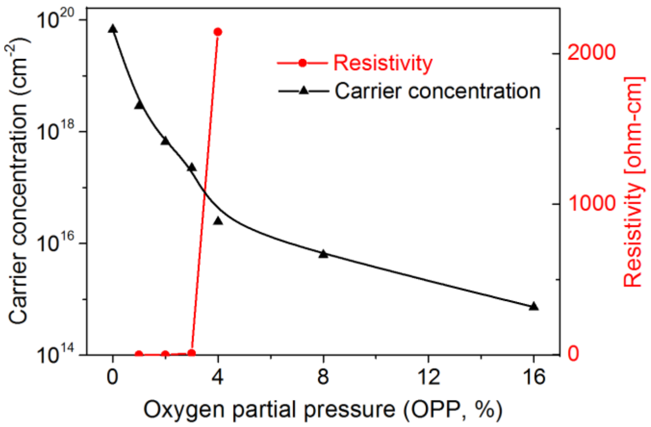

(b)

OPP	I_{on} (A)	I_{off} (A)	I_{on}/I_{off}	Threshold voltage (V)	Mobility (cm^2/Vs)
0	3.1×10^{-3}				
1	6.3×10^{-4}	1.8×10^{-9}	4.5×10^{5}	6.4	15.67
2	1.1×10^{-4}	6.2×10^{-10}	4.9×10^{5}	9.7	6.38
3	4.4×10^{-5}	5.6×10^{-11}	8.8×10^{5}	11.0	2.53
4	1.2×10^{-5}	7.3×10^{-11}	1.6×10^{5}	11.4	0.52
8	1.7×10^{-7}	8.6×10^{-12}	1.9×10^{4}	16.3	0.02
16		8.0×10^{-13}			

Figure S4. (a) The carrier concentration and resistivity of the IGZO film sputtered at different oxygen partial pressures (OPP). (b) A comparison of the transistor parameters, including the on-current, off-current, on/off ratio, threshold voltage and mobility of the IGZO TFTs, in which the IGZO film was sputtered at different OPP values.

The IGZO active layer was deposited at different oxygen partial pressures (OPP) varying from 0 to 16 while sputtering. A Hall measurement was conducted to calculate the carrier concentration and the resistivity of the IGZO film. At OPP=0, the IGZO film revealed a very high carrier concentration due to the high oxygen vacancies within the film, and therefore, it exhibited metallic-like behavior with a low resistance, as shown in Fig. S4a. In contrast, for the other extreme case (OPP=16), the oxygen vacancies were completely filled using the excessive oxygen flow, resulting in a low carrier concentration along with a very high resistance. Therefore, the IGZO film acted as an insulator. We found that the carrier

concentration decreased gradually from 10^{20} to 10^{14} cm^{-3}. Fig. S4b lists the quantitative values of the transistor performance including the on current (I_{on}), off current (I_{off}), on/off ratio ($I_{on/off}$), threshold voltage and mobility with regard to different OPP values.

S5. Hysteresis:

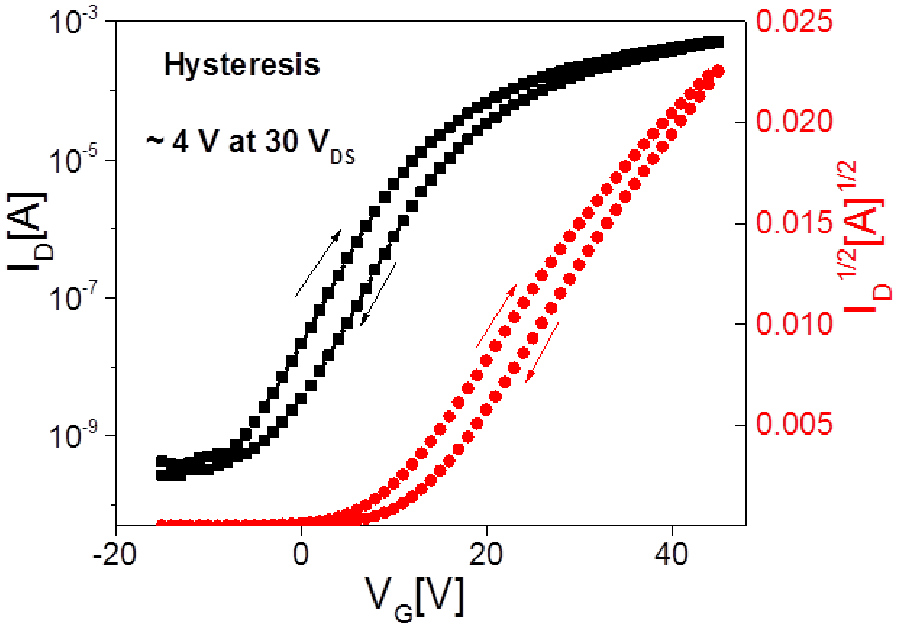

Figure S5. Transfer characteristics, swept in both directions, of the bottom-gated IGZO TFT with a hysteresis value of ~ 4 V at 30 V drain bias.

The bottom-gated IGZO TFT was measured under a dual sweep as shown in Fig. S5. The device demonstrated a hysteresis value of ~ 4 V at 30 V drain bias and the difference in voltage of ~ 4 V between the forward sweep and reverse sweep is due to the shallow traps at the interface between the SA7/IGZO layers. *B.G. Son et al.* reported that their bottom-gated IZO TFTs suffered from large hysteresis (approximately 10 V) due to high defect trap density between IZO/PVA-co-PMMA interface, which is caused by the energetic ion bombardment effect during sputtering.[6] Our bottom-gated IGZO TFTs having the SA7 organic gate dielectric layer exhibited reasonable hysteresis value, indicating a good interface quality.

S6. Comparison of interface trap states (*Nss*) of the SA7-based TFT and PMMA-based TFTs:

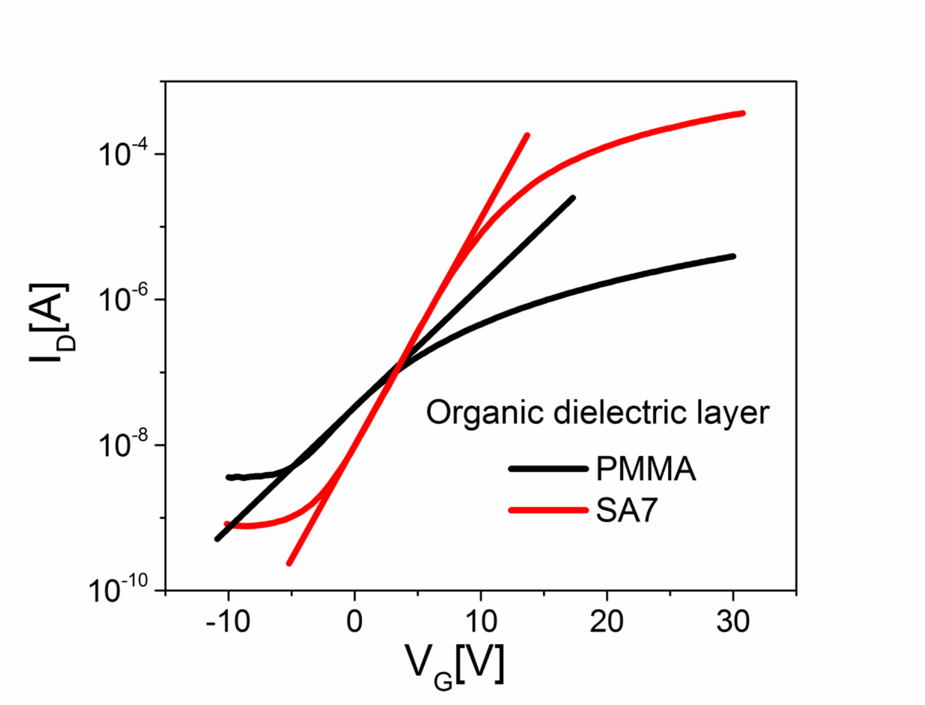

Figure S6. Transfer characteristics of IGZO TFTs with different organic dielectric layers such as PMMA and SA7.

Fig. S6 demonstrates the transfer characteristics of SA7-based TFT and PMMA-based TFT, from which we can calculate the subthreshold slope (SS $=\left[\frac{dV_g}{d\log(I_d)}\right]$). The PMMA-based TFT shows very high subthreshold swing (SS) value (~ 6.5 V/dec) in comparision with the SA7-based TFT (~ 3 V/dec). The number of interface trap density (N_{SS}) can be calculated using a given formula of $N_{SS} = \left[\frac{SS\cdot\log(e)}{KT/q} - 1\right]\frac{C_{OX}}{\mathrm{q}}$,[7-10] where k is Boltzmann coefficient, T is temperature in Kelvin, q is electron charge and C_{OX} is gate oxide capacitance. The results demonstrated that the SA7-based TFT exhibited a lower interface trap density (3.4 x 10^{11} cm^{-2}ev^{-1}) than that of PMMA-based TFT (2.7 x 10^{12} cm^{-2}ev^{-1}). This may be due to the smoother surface and better interface interaction between the IGZO film and the SA7 layer.

S7. Bending tester:

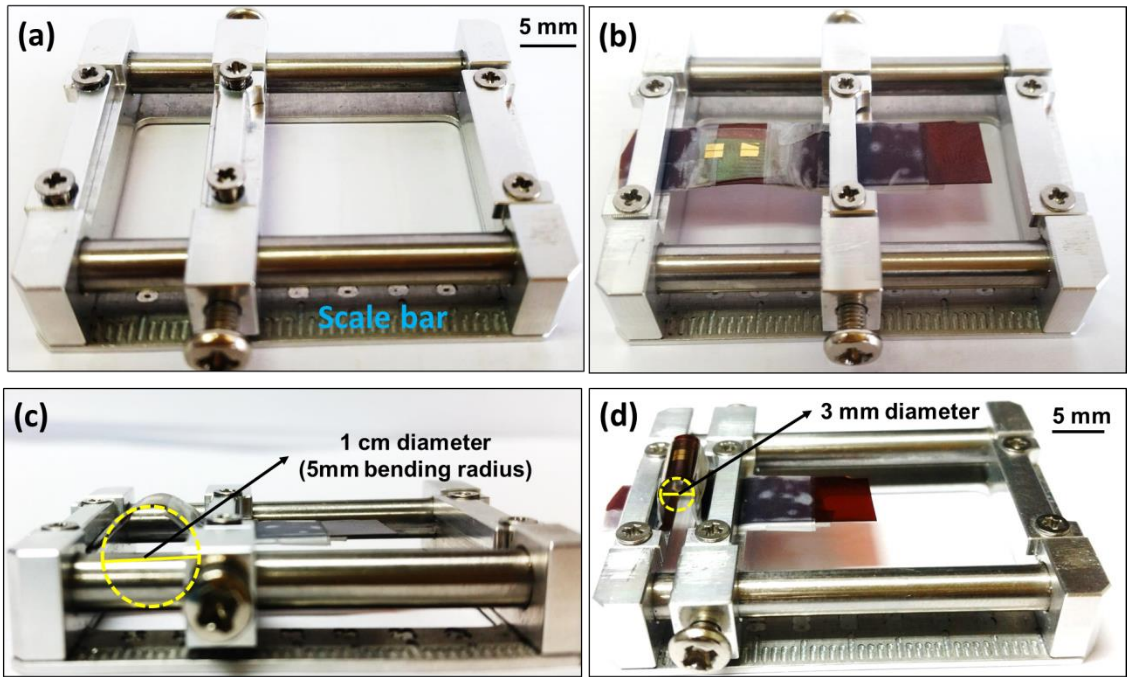

Figure. S7. (a) An image of bending tester. The flexible PI substrate having the IGZO TFTs is placed on the bending tester (b) at pristine condition, and while being bent along channel length direction (c) at 5 mm radius and (d) 1.5 mm radius

The flexible PI substrate having the IGZO TFT is placed on the bending tester as shown in Fig. S7b, in which one corner of the TFT substrate is fixed and the other corner can be moved to control the bending radius. The bending radius is the radius of the yellow dotted circle from the lateral digital view (Fig. S7c, the radius of substrate curvature). We took the digital image under bending at the bending radius of 5 mm and 1.5 mm (Fig. S7c and S7d).

S8. Transfer and output characteristics of TFTs measured while being bent:

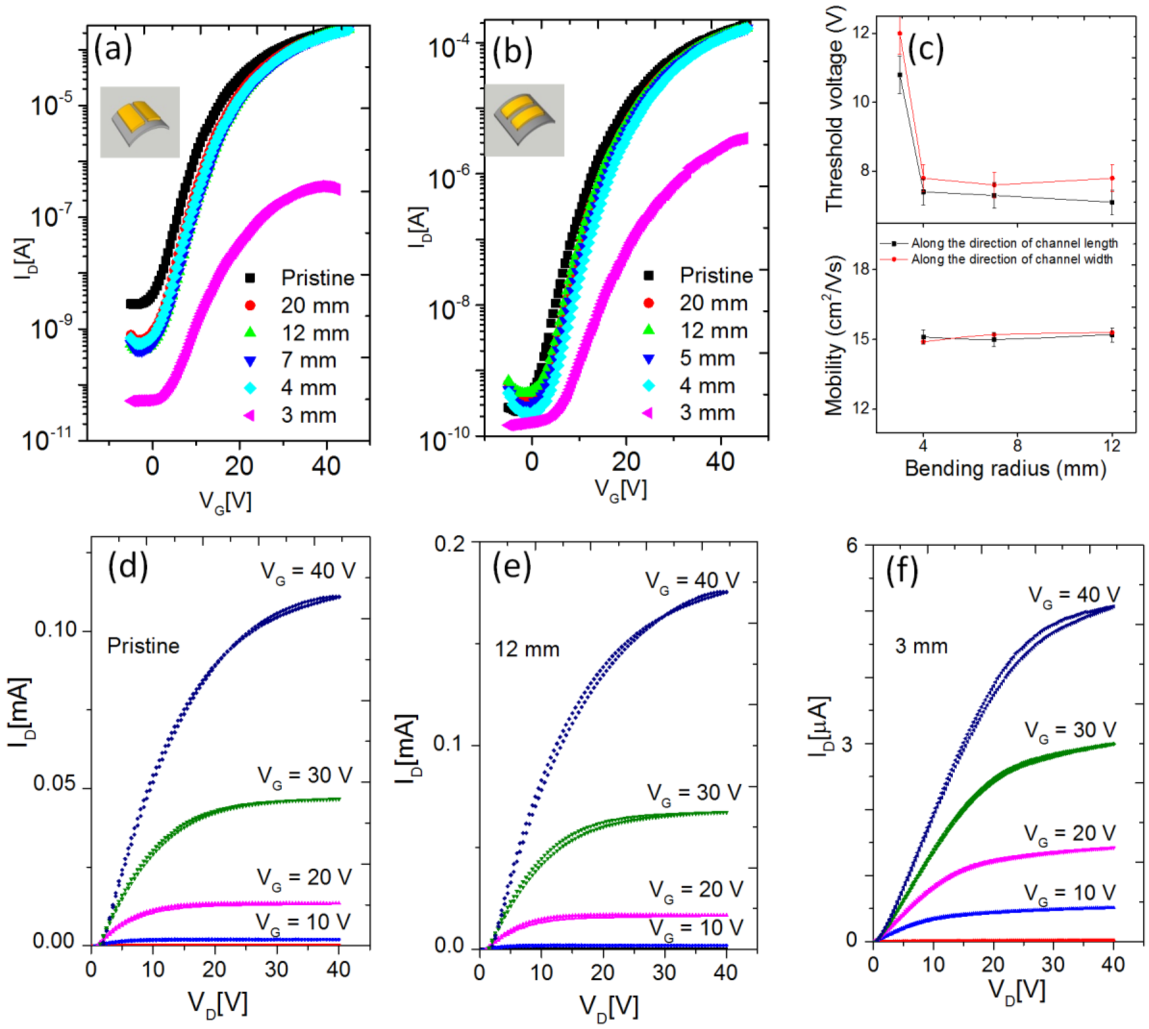

Figure S8. The transfer characteristics of IGZO TFTs while being bent along (a) the channel width direction and (b) the channel length direction at different bending radii. (c) The mobility and threshold voltage for both devices with respect to the bending radii. The output characteristics of IGZO TFT (a) at pristine flat state, and while being bent along the channel width direction at (b) 12 mm bending radius and (c) 3 mm bending radius.

S9. Optical images of the channel region before and after bending along the channel width:

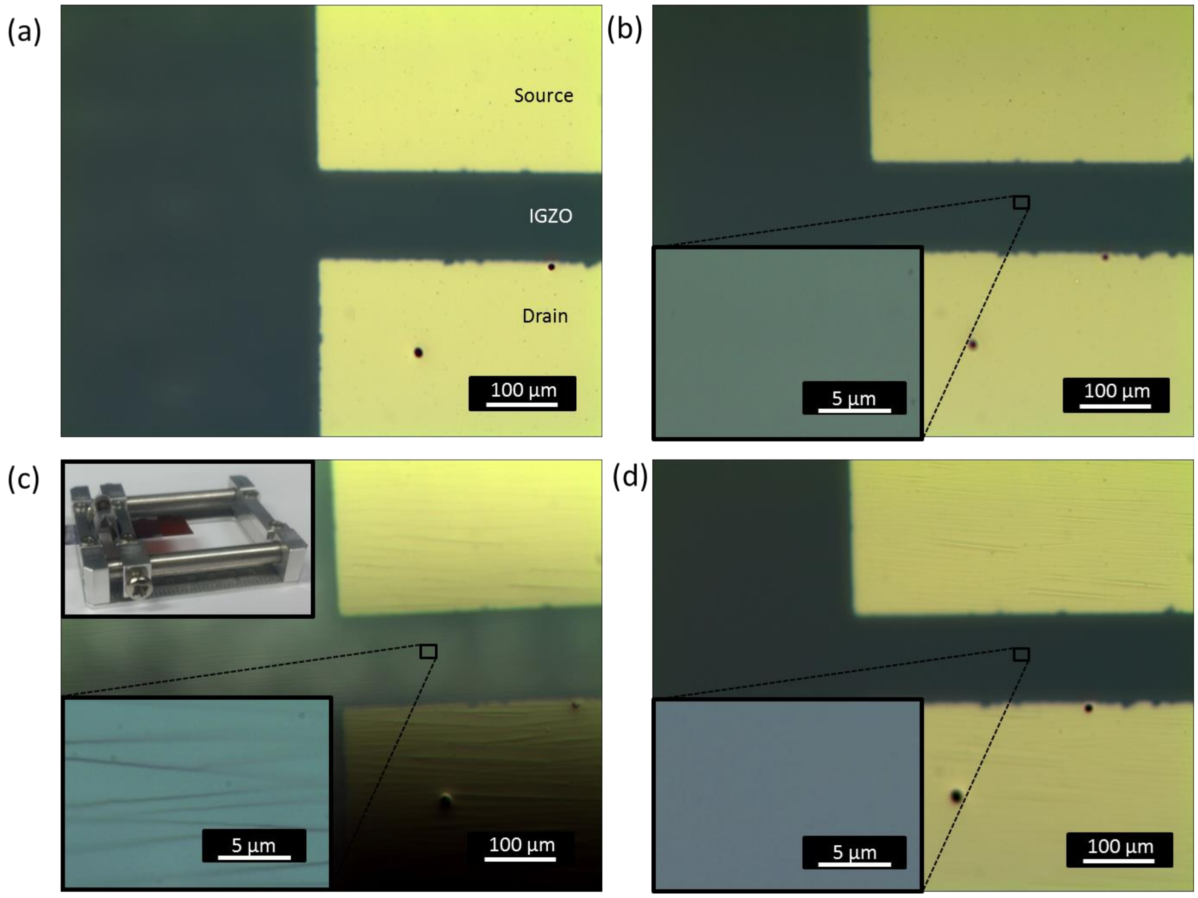

Figure S9. The Optical microscope images of the channel region during bending along the channel width direction under different conditions: (a) flat, while being bent at (b) a 3 mm and (c) a 1.5 mm bending radius, and (d) after releasing the bending stress. Nanoscale cracks run perpendicular to the channel length direction (left bottom inset of Fig. S9c). The insets in Fig. (b), (c) and (d) show the 100 x magnified image of the channel region. The left top inset of Fig. S9c shows the real image of a sample bent at a bending radius of 1.5 mm.

S10. Optical image of channel region before and after bending along the channel length:

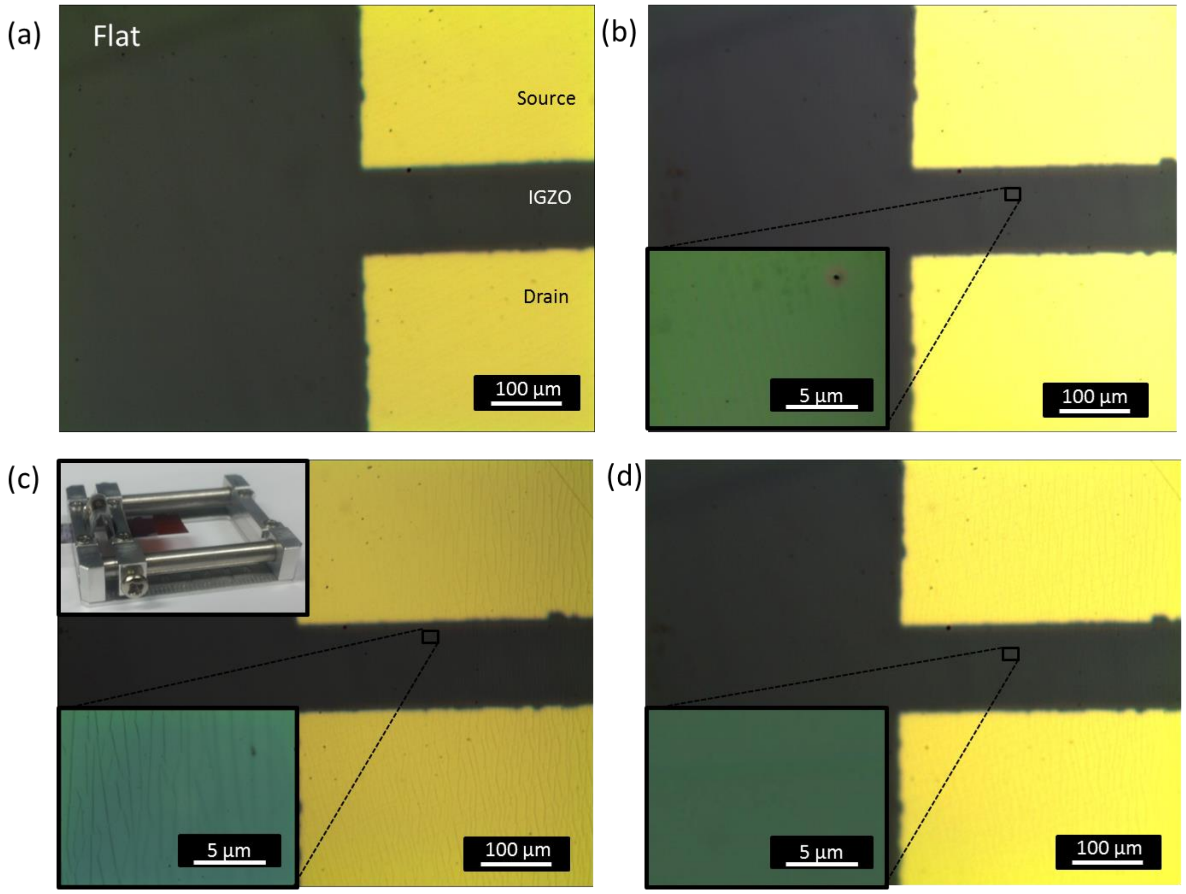

Figure S10. The optical microscope image of the channel region during bending along the channel length direction under different conditions: (a) flat, while being bent at (b) a 3 mm and (c) a 1.5 mm bending radius, and (d) after releasing the bending stress. Nanoscale cracks run parallel to the channel length direction (left bottom inset of Fig. S10c). The insets in the Fig. (b), (c) and (d) show the 100 x magnified image of the channel region. The left top inset of Fig. S10c shows the real image of a sample bent at a bending radius of 1.5 mm.

S11. Optical images of channel region underwent different bending cycles along the channel length direction at 1.5 mm bending radius:

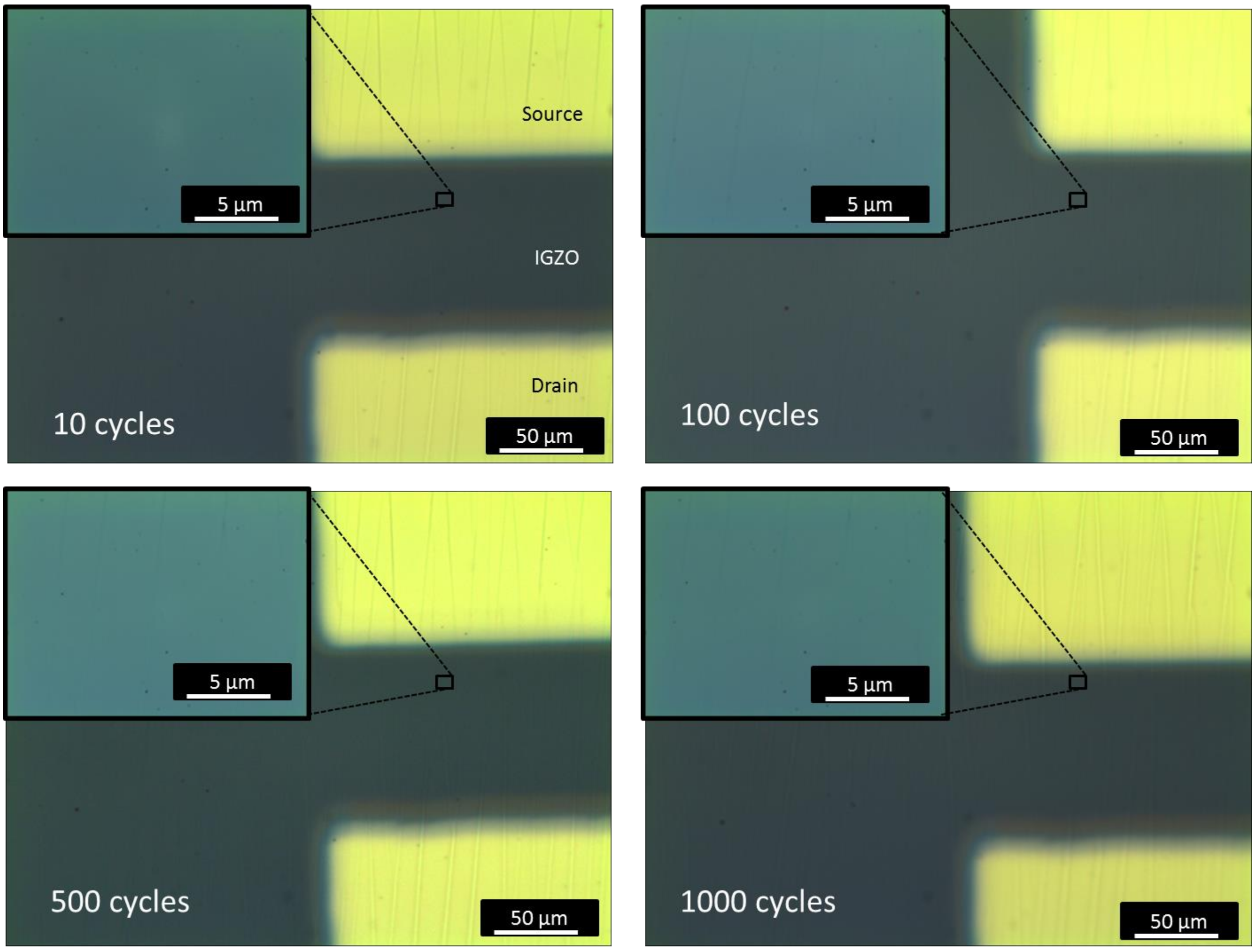

Figure S11. Optical microscope images of the channel region, subjected to various bending cycles (from 10 to 1000 cycles) along the channel length direction at a 1.5 mm bending radius. The insets show the 100 x magnified images of the channel region. The image was taken after releasing the bending stress.

S12. Bending test of IGZO TFT inside a glove box:

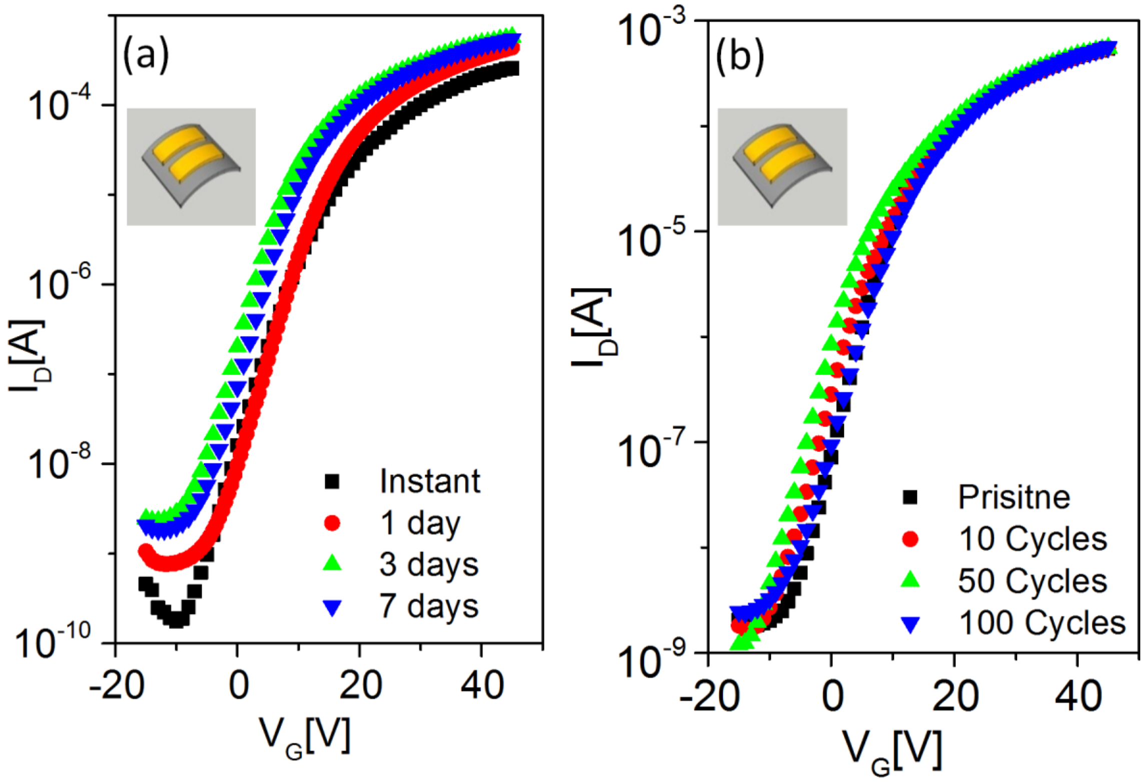

Figure S12. (a) The transfer characteristics of the IGZO TFT at various time intervals measured inside a glove box. Prior to the measurements, the transistor was subjected to 1000 bending cycles at a 1.5 mm bending radius along the channel length direction. The transfer characteristics returned to their initial I_{off} current level after three days. (b) The transfer characteristics of the IGZO TFT subjected to 100 bending cycles along the channel length direction at a 1.5 mm radius inside the glove box exhibited a negligible variation in the transfer characteristics.

S13. Stability test of IGZO TFT inside a glove box:

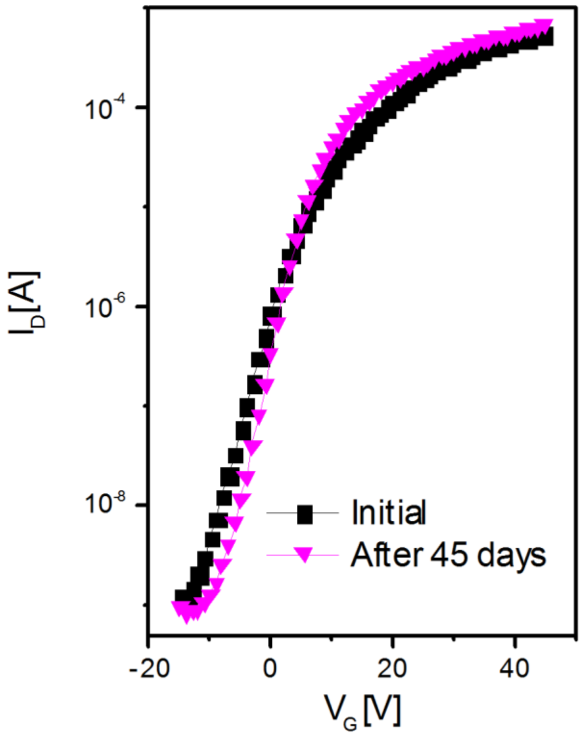

Figure S13. The stability test: the transistor was stored inside a glove box (nitrogen atmosphere). It underwent 100 bending cycles at a 1.5 mm bending radius inside the glove box. The device revealed no significant variation in its electrical behavior even after 45 days.

S14. Fabrication of a flexible IGZO TFT and separation from the PDMS/glass rigid substrate:

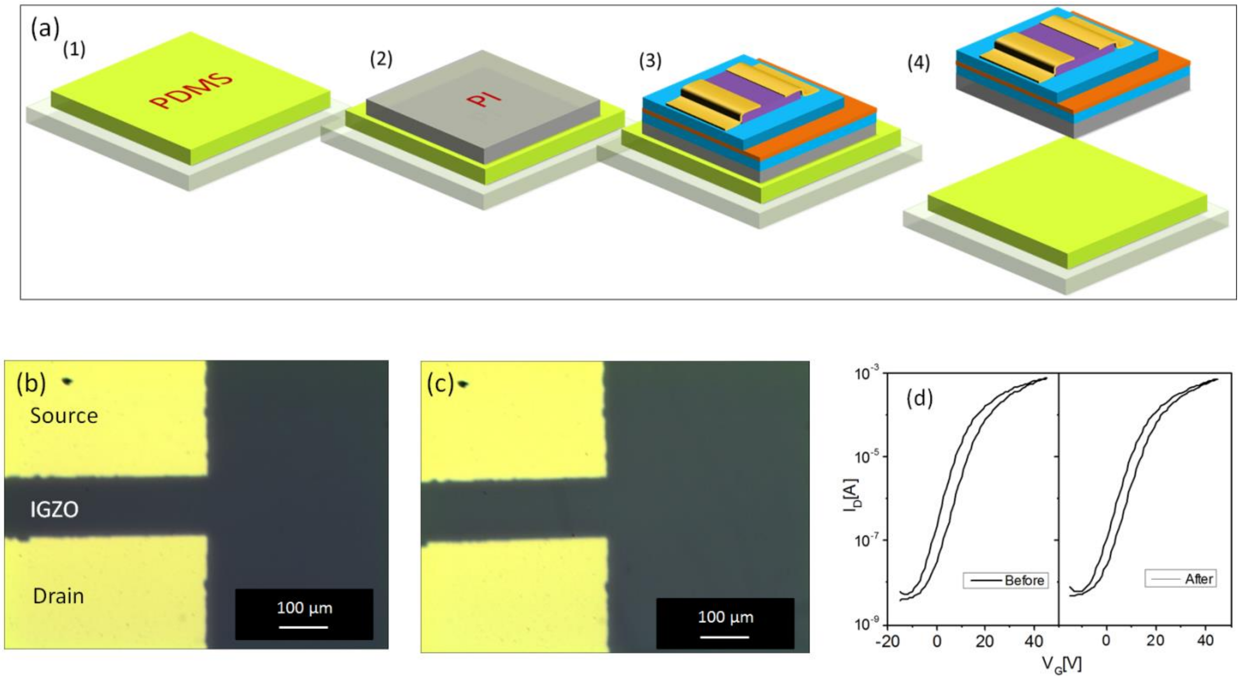

Figure S14. (a) (1-4) A schematic of the fabrication of a bottom-gated IGZO TFT on a PI substrate. Optical microscope images of the top surface of a IGZO TFT (b) before and (c) after detachment from the supporting PDMS/glass substrate and (d) their respective transfer characteristics.

Fig. S14 demonstrates the schematic of the fabrication process of bottom-gated IGZO TFT. To make the fabrication process plausible, we attached the PI substrate to a rigid glass substrate via PDMS, as shown in Fig. S14a. The whole TFT was fabricated on top of the PI substrate, Fig. S14a3. Further, the TFT along with the PI substrate was peeled off the supporting PDMS/glass substrate at the interface between the PI and PDMS with a small force, as demonstrated in Fig. S14a4. Optical image revealed that there was no noticeable damage to the TFT after separation, as shown in Fig. S14b and S14c. In addition, there is no change in the transfer characteristics after separation, which confirms no damage to the IGZO TFT during the separation (Fig. S14d).

References:

1 Duan, G. *et al.* Preparation and characterization of mesoporous zirconia made by using a poly (methyl methacrylate) template. *Nanoscale Res. Lett.* 3, 118-122, doi:10.1007/s11671-008-9123-7 (2008).

2 Sugumaran, S. *et al.* Novel hybrid PVA-InZnO transparent thin films and sandwich capacitor structure by dip coating method: preparation and characterizations. *RSC Adv.* 5, 10599-10610, doi:10.1039/c4ra14817g (2015).

3 Khare, P., Yadav, A., Ramkumar, J. & Verma, N. Microchannel-embedded metal–carbon–polymer nanocomposite as a novel support for chitosan for efficient removal of hexavalent chromium from water under dynamic conditions. *Chem. Eng. J.* 293, 44-54, doi:10.1016/j.cej.2016.02.049 (2016).

4 Hosseini, S. H., Noushin Ezzati, S. & Askari, M. Synthesis, characterization and X-ray shielding properties of polypyrrole/lead nanocomposites. *Polym. Adv. Technol.* 26, 561-568, doi:10.1002/pat.3486 (2015).

5 Singhal, A. *et al.* UV-shielding transparent PMMA/In_2O_3 nanocomposite films based on In_2O_3 nanoparticles. *RSC Adv.* 3, 20913-20921, doi:10.1039/c3ra42244e (2013).

6 Son, B.-G., Je, S. Y., Kim, H. J. & Jeong, J. K. Modification of a polymer gate insulator by zirconium oxide doping for low temperature, high performance indium zinc oxide transistors. *RSC Adv.* 4, 45742-45748, doi:10.1039/c4ra08548e (2014).

7 Xu, Y. *et al.* On the origin of improved charge transport in double-gate In_20_3;Ga_20_3;Zn_20_3;O thin-film transistors: A low-frequency noise perspective. *IEEE Electron Device Lett.* 36, 1040-1043, doi:10.1109/led.2015.2467164 (2015).

8 Unni, K. N. N., Dabos-Seignon, S. & Nunzi, J.-M. Influence of the polymer dielectric characteristics on the performance of a quaterthiophene organic field-effect transistor. *J. Mater. Sci.* 41, 317-322, doi:10.1007/s10853-005-2331-y (2006).

9 Chiu, C. J., Chang, S. P. & Chang, S. J. High-performance a-IGZO thin-film transistor using Ta_2O_5 gate dielectric. *IEEE Electron Device Lett.* 31, 1245-1247, doi:10.1109/led.2010.2066951 (2010).

10 Chang, Y. G. *et al.* Trap density of states measured by photon probe on amorphous-InGaZnO thin-film transistors. *IEEE Electron Device Lett.* 32, 336-338, doi:10.1109/led.2010.2102739 (2011).
